# Risk factors for new antidepressant use after surgery in Sweden: a nationwide, observational cohort study

**DOI:** 10.1016/j.bjao.2023.100218

**Published:** 2023-07-21

**Authors:** Matilda Widaeus, Daniel Hertzberg, Linn Hallqvist, Max Bell

**Affiliations:** 1 Department of Perioperative Medicine and Intensive Care, Karolinska University Hospital, Stockholm, Sweden; 2 Department of Physiology and Pharmacology, Karolinska Institutet, Stockholm, Sweden

**Keywords:** depression, epidemiology, postoperative, surgery

## Abstract

**Background:**

Whilst somatic complications after major surgery are being increasingly investigated, the research field has scarce data on psychiatric outcomes such as postoperative depression. This study evaluates the impact of patient and surgical factors on the risk of depression after surgery using the proxy measure of prescribed and collected antidepressants.

**Methods:**

An observational, registry-based, national multicentre cohort study of individuals ≥18 yr of age who underwent noncardiac surgery between 2007 and 2014. Exclusion criteria included history of antidepressant use defined by collection of a prescription within 5 yr before surgery. Participants were identified using a surgical database from 23 Swedish hospitals and data were linked to National Board of Health and Welfare registers for collection of prescribed antidepressants. Descriptive statistics were used for baseline data and logistic regression for predictive factors.

**Results:**

Of 223 617 patients, 4.9% had a new prescription of antidepressants collected 31–365 days after surgery. Antidepressant prescription was associated with increasing age, female sex, and more comorbidities. The incidence of antidepressant prescription was highest after neurosurgery, vascular, and thoracic surgery. Affective and anxiety disorders were risk factors. In the whole cohort and within the aforementioned surgical subtypes, acute and cancer surgery increased the risk of antidepressant prescription.

**Conclusions:**

This study brings novel insights to the epidemiology of postoperative antidepressant treatment in antidepressant-naive patients. One in 20 postoperative patients are prescribed antidepressants but with knowledge of risk factors, interventional strategies can be tested.

With >300 million surgical procedures performed each year, perioperative morbidity is a heavy societal burden.^1^ Many studies have highlighted cardiac,^1^ renal,^2^ pulmonary,^3^ and neurological complications^4^ and compound metrics of outcome.^5^ Postoperative complications, if survived, may have a major impact on function and quality of life.^6^

Although somatic complications after major surgery are being increasingly investigated, the research field has scarce data on psychological outcomes such as postoperative depression. Most studies use specific surgical cohorts. These include studies of meningioma surgery^7^ and glioma patients^8^; both with an elevated risk of postoperative depression. In contrast, no significant difference in antidepressant use was found after vestibular schwannoma surgery.^9^ When comparing spinal surgery to coronary artery bypass grafting, hysterectomy, cholecystectomy, chronic obstructive pulmonary disease, congestive heart failure exacerbation, and uncomplicated vaginal delivery, the former had the highest risk for postoperative depression.^10^

The aetiology of major depressive disorder (MDD) is poorly understood, and diagnosis is difficult.^11^^,^^12^ Many who experience a first episode of MDD will go on to suffer from recurrent episodes.^13^^,^^14^ When compared with equally disabling somatic disorders, treatment for depression is less prevalent, indicating under-treatment of the condition.^15^ Additionally, individuals with MDD are at increased risk of cardiovascular disease and all-cause mortality.^16^

The present study sought to investigate the impact of patient and surgical factors on risk of postoperative depression in patients undergoing major noncardiac surgery. Surgery may be considered to be a stressful event, potentially triggering depression. Perioperative care may also present an opportunity for formal depression screening. The surrogate marker for depression was new antidepressant drug use. A large national surgical cohort was used and merged with the National Patient Register (NPR), the Swedish Prescribed Drug Register (SPDR), and the Cause of Death Registry (all supplied by the Swedish National Board of Health and Welfare).

## Methods

### Study design and participants

**Fig 1:**
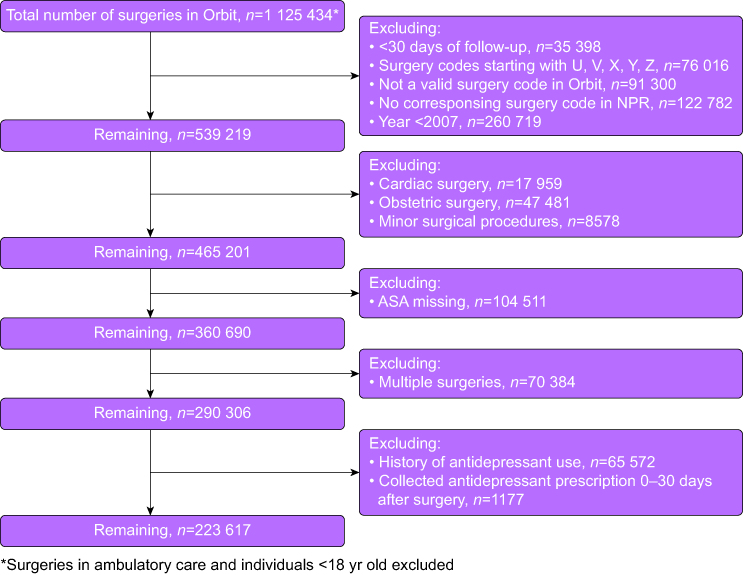
Exclusion criteria. NPR, National Patient Register.

This was an observational, registry-based, multicentre cohort study. The cohort has been described in detail previously.^5^^,^^17^ The study was approved by the Regional Ethics Committee of Stockholm, Sweden (2014/1306-31/3), which waived the need for informed consent from participants. The study population consisted of individuals ≥18 yr of age undergoing surgery between 1 January 2007 and 31 December 2014. Exclusion criteria are shown in the participant flowchart (Fig. 1) and includes history of antidepressant use defined by collection of at least one prescription during 5 yr before the date of surgery. Ambulatory, cardiac, obstetric, and minor operations and patients with missing American Society of Anesthesiologists (ASA) physical status classification were excluded. In cases of participants undergoing multiple operations during the study period, all but the first procedure was excluded. Furthermore, procedures lacking valid surgery codes in the Orbit surgical planning system or corresponding surgery codes in the NPR, patients with <30 days of follow-up and procedures with surgery codes starting with U, V, X, Y or Z were excluded (at the time, these codes represented minor procedures including gastroscopy, etc.). Antidepressants were defined as all preparations with Anatomic Therapeutic Chemical classification system (ATC) Code N06A.

### Data sources

The data were registered prospectively in a total of 23 Swedish hospitals of university, county, and district level between 1999 and 2015.

#### Orbit surgical planning system

Identification of the study population was through Orbit, a surgical planning software. Orbit includes Swedish personal identity number (PIN), patient characteristics, ASA physical status classification, type, date and duration of anaesthesia and surgery. NOMESCO classification^18^ was used to subgroup all procedures into 16 categories; neuro, endocrine, ophthalmic, ear, nose and throat, oral and maxillofacial, cardiac, vascular, thoracic, breast, gastrointestinal, urological, gynaecological, obstetric, orthopaedic, dermatological, and minor surgery.

#### The National Board of Health and Welfare Registers

Surgical records were linked to the NPR, the SPDR, and the Swedish Cause of Death Register using the PIN assigned to all Swedish residents at birth or immigration.^19^

#### The National Patient Register

The NPR contains geographical, administrative, medical, and personal data.^20^ Participation in the register is mandatory for all Swedish county councils.^21^ We based comorbidity data on ICD codes, detailed in Supplementary Table S1.

#### The Swedish Prescribed Drug Register

Established in 2005, SPDR contains information on all *collected* drug prescriptions linked to the PIN. Pre- and postoperative collected drug prescriptions were captured.

#### The Swedish Cause of Death Register

The Swedish Cause of Death Register has information on virtually all deaths in Sweden since 1952,^22^ including Swedish individuals who die abroad, and deaths of non-Swedish residents (since 2012).^22^

#### Primary outcome

Using this surgical cohort, we identified all patients undergoing surgery, alive within the first year after surgery, who had not collected a prescription for antidepressant in the 5 yr before the index surgery. The primary outcome was new antidepressant use defined by the collection of at least one antidepressant prescription between 31 and 365 days after surgery. Collection of prescribed antidepressant drugs, as characterised by the ATC code, was used as a surrogate for depression, instead of the ICD code, since multiple studies have suggested under-reporting of depression diagnosis in the perioperative setting.^23^, ^24^, ^25^

#### Rationale for exclusions

I. Patients who were discharged and collected a prescription for antidepressants within a month of discharge (day 0–30)—these patients were most likely depressed before the surgical event.
II. Patients who had been prescribed and collected antidepressants within the previous 5 yr and therefore very likely to continue on antidepressant medication, thus not contributing information to clinicians regarding potentially modifiable factors for new onset postoperative depression.
III. Those who died within a year of surgery, as these patients were not exposed to the 1-yr risk period that we defined for the development of postoperative depression.
IV. Patients who start antidepressant drugs in hospital for similar reasons to excluding patients prescribed antidepressants within 30 days of discharge (it is very uncommon for patients to start antidepressant drugs in the hospital).

In the supplementary materials, we show all analyses (Supplementary Tables S2–S5) without exclusions of the patients with collection of antidepressants within the first month after surgery.

### Statistical methods

Statistical analyses were performed using Stata version 15 (Stata Corp, College Station, TX, USA). Categorical data are presented as numbers and percentages, age was the only continuous variable and is presented as the mean and standard deviation. Dichotomous and categorical variables were analysed using Pearson's χ^2^ test or Fischer's exact test and age data were analysed using analysis of variance (anova).

Logistic regression was used to identify variables associated with antidepressant use. First, we identified types of surgery that were associated with increased risk of antidepressant use in univariate analyses defined as an odds ratio >1.2 and a *P*-value <0.05. Secondly, we identified background characteristics and surgery characteristics (non-elective surgery, cancer surgery) associated with antidepressant use *within* the high-risk surgery types identified earlier. For this analysis all variables on background characteristics, non-elective surgery, and cancer surgery, were eligible for inclusion in a multivariable model except ASA classification. ASA classification was not eligible since this variable includes other background characteristics and risks collinearity in the analysis. Eligible variables that were associated with the outcome with a *P*-value <0.05 in univariate logistic regression were included in a multivariable model for a subsequently manual backward selection procedure. Variables no longer statistically associated (*P*-value ≥0.05) were excluded. Furthermore, as predefined, variables with an odds ratio <1.2 or >0.8 were also excluded because of their small clinical impact. The procedure was repeated for each high-risk surgery type.

The analysis of risk factors for antidepressant use within the high-risk surgery types was repeated but this time the eligible variables were age, sex, ASA classification, non-elective surgery, and cancer surgery. ASA classification was in this analysis as a score for comorbidities. The Wald test was used to analyse the overall association with ASA classification and antidepressant use.

There was a non-linear association with age and antidepressant use in all analyses. Age was therefore modelled using restricted cubic splines with three knots. The Wald test was used to analyse the splines association with antidepressant use.

Significant, complex interactions between patient characteristics and different surgical types detected in the cohort precluded planned analyses including all patients and surgical types.

## Results

We identified 1 125 434 eligible patients who underwent surgery at any of the 23 hospitals in Sweden from January 2007 to December 2014; after exclusions, data on 223 617 patients were available for complete analysis (Fig. 1).

Table 1 presents differences between post-surgery antidepressant users and non-users. The mean age was 59.5 yr and 52% of the population were women. We found a 4.9% incidence of postoperative collection of antidepressant medication. The postoperative antidepressant users were older, more often women, had more comorbid conditions and following that, higher ASA classification. Acute surgery was associated with postoperative antidepressant use. We found no temporal differences between the years 2007 and 2010 compared with 2011–4.

**Table 1:** Patient and perioperative characteristics in relation to collection of antidepressant medication after major surgery. Abbreviations: ASA = American Society of Anesthesiologists, SD = Standard Deviation. ∗ANOVA. ∗∗ Pearson’s Chi-squared test.

	Collected antidepressant prescription 31–365 days post-surgery			
	All (*n* = 210668)	No (*n* =200865)	Yes (*n* = 9803)	*P*-value
**Background characteristics**				
Age, yr, mean (SD)	58.5 (18.9)	58.4 (18.9)	60.5 (19.1)	<0.001^∗^
Female sex, *n* (%)	109780 (52.1)	104204 (51.9)	5576 (56.9)	<0.001^∗∗^
ASA Classification, *n* (%)				<0.001^∗∗^
ASA1	72856 (34.6)	70603 (35.1)	2253 (23.0)	
ASA2	92762 (44.0)	88753 (44.2)	4009 (40.9)	
ASA3	42536 (20.2)	39378 (19.6)	3158 (32.2)	
ASA4	2514 (1.2)	2131 (1.1)	383 (3.9)	
Heart disease*, n* (%)	51766 (24.6)	48875 (24.3)	2891 (29.)	<0.001^∗∗^
Chronic kidney disease*, n* (%)	3581 (1.7)	3377 (1.7)	204 (2.1)	0.003^∗∗^
Diabetes Mellitus*, n* (%)	11017 (5.2)	10276 (5.1)	741 (7.6)	<0.001^∗∗^
Peripheral vascular disease*, n* (%)	7747 (3.7)	7182 (3.6)	565 (5.8)	<0.001^∗∗^
Cerebrovascular disease*, n* (%)	6809 (3.2)	6253 (3.1)	556 (5.7)	<0.001^∗∗^
Cognitive disease*, n* (%)	2097 (1.0)	1833 (0.9)	264 (2.7)	<0.001^∗∗^
Substance abuse disorder*, n* (%)	2011 (1.0)	1801 (0.9)	210 (2.1)	<0.001^∗∗^
Miscellaneous psychiatric disorders, *n* (%)	3276 (1.6)	3042 (1.5)	234 (2.4)	<0.001^∗∗^
Affective disorders*, n* (%)	1324 (0.6)	1170 (0.6)	154 (1.6)	<0.001^∗∗^
Anxiety disorders*, n* (%)	3101 (1.5)	2792 (1.4)	309 (3.2)	<0.001^∗∗^
Chronic obstructive pulmonary disease*, n* (%)	4386 (2.1)	4048 (2.0)	338 (3.4)	<0.001^∗∗^
Year of surgery 2011–2014 (Compared to 2007–2010)*, n* (%)	122098 (58.0)			0.29^∗∗^
Type of surgery, *n* (%)		116366 (57.9)	5732 (58.5)	
Non-elective surgery*, n* (%)	60271 (28.6)	56902 (28.3)	3369 (34.4)	<0.001^∗∗^
Cancer surgery*, n* (%)	42776 (20.3)	40335 (20.1)	2441 (24.9)	<0.001^∗∗^
Neuro*, n* (%)	14288 (6.8)	12807 (6.4)	1481 (15.1)	<0.001^∗∗^
Endocrine*, n* (%)	6702 (3.2)	6452 (3.2)	250 (2.6)	<0.001^∗∗^
Ophthalmic*, n* (%)	2923 (1.4)	2843 (1.4)	80 (0.8)	<0.001^∗∗^
Ear Nose and Throat*, n* (%)	6219 (3.0)	6056 (3.0)	163 (1.7)	<0.001^∗∗^
Oral and maxillofacial*, n* (%)	8510 (4.0)	8198 (4.1)	312 (3.2)	<0.001^∗∗^
Thoracic (not including cardiac surgery)*, n* (%)	2142 (1.0)	1992 (1.0)	150 (1.5)	<0.001^∗∗^
Breast*, n* (%)	10775 (5.1)	10221 (5.1)	554 (5.7)	0.014^∗∗^
Abdominal*, n* (%)	41058 (19.5)	39367 (19.6)	1691 (17.2)	<0.001^∗∗^
Urological*, n* (%)	23121 (11.0)	22132 (11.0)	989 (10.1)	0.004^∗∗^
Gynaecological*, n* (%)	16510 (7.8)	15852 (7.9)	658 (6.7)	<0.001^∗∗^
Orthopaedic*, n* (%)	64097 (30.4)	61432 (30.6)	2665 (27.2)	<0.001^∗∗^
Vascular*, n* (%)	8632 (4.1)	8089 (4.0)	543 (5.5)	<0.001^∗∗^
Dermatological*, n* (%)	5691 (2.7)	5424 (2.7)	267 (2.7)	0.89^∗∗^

Table 2 details data for the subgroups with the highest antidepressant collection: neurosurgery, vascular surgery, and thoracic surgery. The incidence of antidepressant use among neurosurgery patients was 10.4% (*P*<0.0001), vascular surgery patients 6.5% (*P*<0.0001), and thoracic surgery patients 6.6% (*P*<0.0001). Female sex, higher ASA classification, and acute surgery were associated with postoperative antidepressant use across all three major surgical subtypes. This was also the case for affective and anxiety disorders, albeit with non-significant increases in some instances.

**Table 2:** Patient and perioperative characteristics in relation to collection of antidepressant medication after major surgery. Abbreviations: ASA = American Society of Anesthesiologists, SD = Standard Deviation. ∗ANOVA. ∗∗ Fisher's exact test.

	Collected antidepressant prescription 31–365 days post-surgery								
	Neurosurgery			Vascular surgery			Thoracic surgery (no heart surgery)		
	No (*n* = 12807)	Yes (*n* = 1481)	*P*-value	No (*n* = 8089)	Yes (*n* = 543)	P-value	No (*n* = 1992)	Yes (*n* = 150)	*P*-value
**Background characteristics**									
Age*,* yr, mean (SD)	57.5 (17.7)	57.5 (16.2)	0.97^∗^	66.4 (14.5)	67.7 (14.8)	0.041^∗^	54.1 (19.4)	59.0 (17.4)	0.003^∗^
Female sex, *n*. (%)	5425 (42.4)	698 (47.1)	<0.001^∗∗^	3098 (38.3)	253 (46.6)	<0.001^∗∗^	760 (38.2)	72 (48.0)	0.017^∗∗^
ASA Classification, *n* (%)			<0.001^∗∗^			<0.001^∗∗^			<0.001^∗∗^
ASA1	2355 (18.4)	147 (9.9)		1228 (15.2)	60 (11.0)		406 (20.4)	10 (6.7)	
ASA2	4903 (38.3)	412 (27.8)		2979 (36.8)	155 (28.5)		740 (37.1)	42 (28.0)	
ASA3	5159 (40.3)	749 (50.6)		3588 (44.4)	294 (54.1)		766 (38.5)	75 (50.0)	
ASA4	390 (3.0)	173 (11.7)		294 (3.6)	34 (6.3)		80 (4.0)	23 (15.3)	
Heart disease*, n* (%)	3251 (25.4)	397 (26.8)	0.24^∗∗^	3839 (47.5%)	311 (57.3)	<0.001^∗∗^	495 (24.8)	40 (26.7)	0.63^∗∗^
Chronic kidney disease*, n* (%)	110 (0.9)	11 (0.7)	0.76^∗∗^	766 (9.5)	42 (7.7)	0.20^∗∗^	25 (1.3)	0 (0.0)	0.41^∗∗^
Diabetes Mellitus*, n* (%)	715 (5.6)	83 (5.6)	0.95^∗∗^	948 (11.7)	89 (16.4)	0.002^∗∗^	106 (5.3)	11 (7.3)	0.27^∗∗^
Peripheral vascular disease*, n* (%)	327 (2.6)	33 (2.2)	0.54^∗∗^	2707 (33.5)	199 (36.6)	0.13^∗∗^	66 (3.3)	5 (3.3)	1.00^∗∗^
Cerebrovascular disease*, n* (%)	1023 (8.0)	132 (8.9)	0.23^∗∗^	688 (8.5)	62 (11.4)	0.022^∗∗^	41 (2.1)	7 (4.7)	0.076^∗∗^
Cognitive disease*, n* (%)	203 (1.6)	29 (2.0)	0.28^∗∗^	66 (0.8)	12 (2.2)	0.003^∗∗^	12 (0.6)	2 (1.3)	0.26^∗∗^
Substance abuse disorder*, n* (%)	168 (1.3)	28 (1.9)	0.076^∗∗^	138 (1.7)	17 (3.1)	0.028^∗∗^	38 (1.9)	4 (2.7)	0.53^∗∗^
Miscellaneous psychiatric disorders, *n* (%)	266 (2.1)	17 (1.1)	0.013^∗∗^	65 (0.8)	5 (0.9)	0.63^∗∗^	50 (2.5)	4 (2.7)	0.79^∗∗^
Affective disorders*, n* (%)	85 (0.7)	22 (1.5)	0.002^∗∗^	47 (0.6)	6 (1.1)	0.15^∗∗^	19 (1.0)	4 (2.7	0.072^∗∗^
Anxiety disorders*, n* (%)	207 (1.6)	31 (2.1)	0.20^∗∗^	87 (1.1)	15 (2.8)	0.002^∗∗^	34 (1.7)	8 (5.3)	0.007^∗∗^
Chronic obstructive pulmonary disease*, n* (%)	227 (1.8)	32 (2.2)	0.30^∗∗^	439 (5.4)	36 (6.6)	0.24^∗∗^	114 (5.7)	14 (9.3)	0.075^∗∗^
Non-elective surgery*, n* (%)	3465 (27.1)	579 (39.1)	<0.001^∗∗^	1337 (16.5)	147 (27.1)	<0.001^∗∗^	516 (25.9)	66 (44.0)	<0.001^∗∗^
Cancer surgery*, n* (%)	1162 (9.1)	148 (10.0)	0.25^∗∗^	2637 (32.6)	132 (24.3)	<0.001^∗∗^	638 (32.0)	49 (32.7)	0.86^∗∗^

Table 3, Table 4 present the variables independently associated with antidepressant use from the multivariable logistic regression analysis; the crude and adjusted odds ratios, again separated by surgical subtype. Worth highlighting is that female neurosurgical patients have a 26% increased risk of postoperative antidepressant use and patients with affective disorders have a more than doubled risk. Acute and cancer surgery in neurosurgical patients was associated with increased antidepressant use. Among vascular surgery patients, female sex, heart disease, and cognitive disease were some of the important risk factors. In thoracic surgery, female sex, anxiety disorders, and acute surgery were the only statistically and clinically significant factors. Table 4 presents the second multivariable analysis where ASA classification represents background diseases.

**Table 3:** Variables independently associated with antidepressant use 31–365 days after major surgery. Multivariable model 1: all characteristics variables in Table 1 eligible for inclusion except ASA classification. ∗The nonlinear association between age and the outcome was modeled using restricted cubic splines. The odds ratios and confidence intervals according to the age categories were calculated based on the spline coefficients. The *P-*value was calculated using Wald’s test. Abbreviations: ASA = American Society of Anesthesiologists, CI = Confidence interval.

	Crude odds ratio (95% CI)	Multivariable adjusted odds ratio (95% CI)
**Neurosurgery**		
Age (yr)^∗^	*P*<0.0001	*P*<0.0001
18	1.0 (Reference)	1.0 (Reference)
30	1.23 (1.13–1.35)	1.25 (1.14–1.36)
40	1.46 (1.25–1.72)	1.49 (1.27–1.75)
50	1.66 (1.34–2.05)	1.69 (1.36–2.10)
60	1.68 (1.33–2.13)	1.71 (1.34–2.17)
70	1.48 (1.18–1.85)	1.47 (1.17–1.85)
80	1.20 (0.97–1.49)	1.17 (0.94–1.45)
90	0.96 (0.76–1.22)	0.92 (0.72–1.16)
Female sex	1.21 (1.09–1.35)	1.26 (1.13–1.40)
Miscellaneous psychiatric disorders	0.55 (0.33–0.90)	0.55 (0.33–0.91)
Affective disorders	2.26 (1.41–3.62)	2.30 (1.42–3.73)
Non-elective surgery	1.73 (1.55–1.93)	1.82 (1.62–2.03)
**Vascular surgery**		
Female sex	1.41 (1.18–1.67)	1.50 (1.26–1.79)
Heart disease	1.48 (1.25–1.77)	1.40 (1.16–1.68)
Diabetes mellitus	1.48 (1.16–1.87)	1.31 (1.02–1.68)
Cognitive disease	2.75 (1.48–5.11)	2.11 (1.11–3.98)
Anxiety disorders	2.61 (1.50–4.55)	2.30 (1.31–4.06)
Substance abuse disorder	1.86 (1.12–3.11)	1.69 (1.00–2.84)
Non-elective surgery	1.87 (1.54–2.29)	1.85 (1.51–2.25)
**Thoracic surgery (not including cardiac surgery)**		
Age (yr)^∗^		
18	1.0 (Reference)	1.0 (Reference)
30	1.27 (0.95–1.69)	1.29 (0.96–1.72)
40	1.53 (0.94–2.50)	1.57 (0.95–2.59)
50	1.81 (0.97–3.38)	1.87 (0.99–3.53)
60	2.07 (1.08–3.99)	2.13 (1.09–4.18)
70	2.29 (1.25–4.19)	2.33 (1.24–4.36)
80	2.48 (1.36–4.52)	2.49 (1.34–4.62)
90	2.69 (1.34–5.38)	2.65 (1.31–5.39)
Female sex	1.50 (1.07–2.09)	1.60 (1.13–2.26)
Anxiety disorders	3.24 (1.47–7.14)	3.33 (1.47–7.53)
Non-elective surgery	2.25 (1.60–3.15)	2.45 (1.73–3.47)

**Table 4:** Variables independently associated with antidepressant use 31–365 days after major surgery. Multivariable model 2: variables age, sex, ASA classification, non-elective surgery, and cancer surgery eligible for inclusion. ∗The nonlinear association between age and the outcome was modeled using restricted cubic splines. The odds ratios and confidence intervals according to the age categories were calculated based on the spline coefficients. The *P-*value was calculated using Wald’s test. Abbreviations: ASA = American Society of Anesthesiologists, CI = Confidence interval.

	Crude odds ratio (95% CI)	Multivariable adjusted odds ratio (95% CI)
**Neurosurgery**		
ASA classification	*P*<0.0001	*P*<0.0001
ASA1	1.0 (Reference)	1.0 (Reference)
ASA2	1.35 (1.11–1.64)	1.47 (1.20–1.80)
ASA3	2.33 (1.94–2.79)	2.42 (2.00–2.93)
ASA4	7.11 (5.56–9.08)	6.60 (5.12–8.52)
Age (yr)^∗^	*P*<0.0001	*P*<0.0001
18	1.0 (Reference)	1.0 (Reference)
30	1.23 (1.13–1.35)	1.16 (1.06–1.27)
40	1.46 (1.25–1.72)	1.31 (1.11–1.54)
50	1.66 (1.34–2.05)	1.40 (1.13–1.74)
60	1.68 (1.33–2.13)	1.35 (1.06–1.72)
70	1.48 (1.18–1.85)	1.12 (0.88–1.41)
80	1.20 (0.97–1.49)	0.85 (0.68–1.07)
90	0.96 (0.76–1.22)	0.65 (0.50–0.83)
Female sex	1.21 (1.09–1.35)	1.25 (1.12–1.40)
Non-elective surgery	1.73 (1.55–1.93)	1.44 (1.28–1.63)
**Vascular surgery**		
ASA classification	*P*<0.0001	*P*<0.0001
ASA1	1.0 (Reference)	1.0 (Reference)
ASA2	1.06 (0.78–1.45)	1.11 (0.82–1.51
ASA3	1.68 (1.26–2.23)	1.72 (1.29–2.30)
ASA4	2.37 (1.53–3.67)	2.25 (1.43–3.53)
Female sex	1.41 (1.18–1.67)	1.55 (1.30–1.85)
Non-elective surgery	1.87 (1.54–2.29)	1.69 (1.38–2.07)
**Thoracic surgery (not including cardiac surgery)**		
ASA classification	*P*<0.0001	*P*<0.0001
ASA1	1.0 (Reference)	1.0 (Reference)
ASA2	2.30 (1.14–4.64)	2.69 (1.32–5.49)
ASA3	3.98 (2.03–7.77)	4.43 (2.25–8.72)
ASA4	11.67 (5.35–25.47)	9.64 (4.38–21.20)
Female sex	1.50 (1.07–2.09)	1.59 (1.12–2.25)
Non-elective surgery	2.25 (1.60–3.15)	2.36 (1.64–3.39)

Supplementary Tables S1–S4 mirror these analyses without exclusions of the patients with collection of antidepressants within the first month after surgery. Very small differences are seen; in vascular surgery patients, heart disease was not a significant risk factor but affective disorders were.

## Discussion

In this large national cohort study of adults undergoing major noncardiac surgery, collection of antidepressant medication among previous non-users occurred in close to one out of 20 patients. Neurosurgery, vascular surgery, and thoracic surgery were identified as high-risk groups. Among these surgery groups, female sex, higher somatic comorbid burden—measured as ASA class, specific, or both comorbidities—and preoperative affective and anxiety disorders were more common in the group treated with antidepressants.

The need to identify surgical patients at risk of anxiety and depression has been identified by multiple research groups. The perioperative process is stressful, mostly because of the surgical trauma. It can lead to anxiety or depression.^26^^,^^27^ Increased anxiety levels are in turn associated with somatic postoperative morbidity and multiple studies show that patients with MDD have an increased prevalence of postoperative infections.^28^, ^29^, ^30^ Data show that depression may negatively impact immunity leading to elevated risk of infections after surgery and tumour growth in cancer patients.^31^

In an observational study of 200 surgical patients, almost half had depression and one-quarter anxiety.^32^ An Irish study compared two exposures: hospitalised patients and patients (hospitalised *and*) undergoing surgery, with controls,^33^ finding an increased incidence of depressive symptoms in both exposed groups. A study of breast cancer surgery evaluated 247 patients postoperatively and after 1 yr; 165 had no depression, 40 had recovered from depression, 24 had incident depression, and 18 had persistent depression.^34^

Most publications emanate from single-centre studies of one type of surgery; examples beside breast cancer^34^ include lower limb amputation,^35^ eye cataract surgery,^36^ and orthognathic surgery.^37^ In contrast, the present study cohort consists of multiple types of major surgery across all adult age groups. This national cohort is much larger, consisting of >200 000 patients from 23 hospitals from all parts of Sweden. Our outcome differed: we chose not to use postoperative *diagnosis* (of depression) and instead used the SPDR to extract data on collected prescriptions of antidepressant drugs. Our findings range from expected to novel: females have a higher incidence of depression and preoperative affective and anxiety disorders are known to be linked to depression, use of antidepressant medication, or both. However, the fact that ASA classification was associated with postoperative antidepressant use has not been shown before. This begs the question, are patients worrying about their health and does this lead to depression? Or does it suggest that people with more access to surgical care also have more access to psychiatric screening? Among patients after cardiac surgery (and after myocardial infarction) anxiety is well documented.^38^ Interestingly, data suggest that cognitive behavioural therapy could be beneficial.^39^ Notably, one study of cardiac surgery demonstrated that an increase in depressive symptoms predicted a decrease in physical functioning but not the other way around.^40^ Even though this study lacks the resolution to determine potential causal pathways, identification of patients at risk of anxiety and depression by healthcare providers is paramount to deliver optimised care. The surgical subtypes and the risk factors we present in this study allow for targeted interventions to be tested. Future studies of interventional strategies are needed, acknowledging that treatment with antidepressant drugs can in many cases be the correct intervention.

This study has limitations. It is observational and presents data with follow-up to 2015, so changes with regards to outcomes during the last 6–7 yr are lacking. We do not have data on pre- or postoperative depression using scales, nor can we detect non-pharmacological therapies and milder disease; instead, we lean heavily on the proxy measure of prescription *and collection* of antidepressants. Importantly, these drug classes are prescribed for several other psychiatric conditions besides depression, even though most prescriptions of antidepressant drugs aim to treat depression. Furthermore, multiple analyses were used in the identification of variables associated with the outcome. When performing multiple tests there is always a risk of including variables not truly associated with the outcome. Data on socioeconomic status are lacking. As in all large registry-based studies, there could be data entry errors. There are also strengths. This is by far the largest cohort studied with regards to antidepressant use as a proxy for postoperative depression. We have avoided minor and ambulatory procedures as these could dilute our cohort. Moreover, by excluding obstetric and cardiac surgery, we avoid postpartum depression and post-*coronary artery bypass graft* (CABG) depression. It is a nationwide cohort covering hospitals of all sizes, ranging from small regional and specialised institutions to large university hospitals. Using the PIN allowed us to merge relevant registers, and specifically, made it possible to extract data on pre- and postoperative collection of prescribed antidepressant drugs.

## Conclusions

New prescription *and collection* of antidepressant drugs among adult patients undergoing major noncardiac surgery is fairly common, especially in neurosurgery, vascular surgery, and thoracic surgery. Acute and cancer procedures were associated with postoperative antidepressant use. The overall incidence was elevated among women, and higher ASA class was a risk factor. Previous diagnosis of affective and anxiety disorder was strongly associated with antidepressant use.

## Authors’ contributions

Conceptualisation of the project, writing and editing the manuscript: all authors

Data curation: MW

Study design: MW, DH

Data analysis: MW, DH, LH

Methodology: DH, MB

Project leading: MB

Read and approved the final manuscript: all authors.

## Declaration of interest

The authors declare that they have no conflicts of interest.
