# Supplementary material for: Risk factors for new antidepressant use after surgery in Sweden: a nationwide, observational cohort study
Source: BJA Open. 2023 Jul 21;7:100218. doi: 10.1016/j.bjao.2023.100218 (PMC10457487; doi:10.1016/j.bjao.2023.100218)
Supplement: Multimedia component 1 [file mmc1.docx]

| **Supplemental Table 1** Patient and perioperative characteristics in relation to collection of antidepressant medication after major surgery | | | | |
| --- | --- | --- | --- | --- |
|  |  | ***Collected antidepressant prescription***  ***0-365 days post-surgery*** | |  |
|  | ***All***  ***(n = 211614)*** | ***No***  ***(n =200865)*** | ***Yes***  ***(n = 10749)*** | ***P-value*** |
| **Background characteristics** |  |  |  |  |
| Age, years, mean ± SD | *58.5 (18.9)* | *58.4 (18.9)* | *60.6 (19.1)* | *<0.001** |
| Female sex, no. (%) | *110282 (52.1%)* | *104204 (51.9%)* | *6078 (56.5%)* | *<0.001*** |
| ASA Classification, no. (%) |  |  |  | *<0.001*** |
| ASA1 | *73070 (34.5%)* | *70603 (35.1%)* | *2467 (23.0%)* |  |
| ASA2 | *93163 (44.0%)* | *88753 (44.2%)* | *4410 (41.0%)* |  |
| ASA3 | *42844 (20.2%)* | *39378 (19.6%)* | *3466 (32.2%)* |  |
| ASA4 | *2537 (1.2%)* | *2131 (1.1%)* | *406 (3.8%)* |  |
| Heart disease, no. (%) | *52064 (24.6%)* | *48875 (24.3%)* | *3189 (29.7%)* | *<0.001*** |
| Chronic kidney disease, no. (%) | *3601 (1.7%)* | *3377 (1.7%)* | *224 (2.1%)* | *0.002*** |
| Diabetes Mellitus, no. (%) | *11085 (5.2%)* | *10276 (5.1%)* | *809 (7.5%)* | *<0.001*** |
| Peripheral vascular disease, no. (%) | *7806 (3.7%)* | *7182 (3.6%)* | *624 (5.8%)* | *<0.001*** |
| Cerebrovascular disease, no. (%) | *6888 (3.3%)* | *6253 (3.1%)* | *635 (5.9%)* | *<0.001*** |
| Cognitive disease, no. (%) | *2126 (1.0%)* | *1833 (0.9%)* | *293 (2.7%)* | *<0.001*** |
| Substance abuse disorder, no. (%) | *2038 (1.0%)* | *1801 (0.9%)* | *237 (2.2%)* | *<0.001*** |
| Miscellaneous psychiatric disorders, no. (%) | *3299 (1.6%)* | *3042 (1.5%)* | *257 (2.4%)* | *<0.001*** |
| Affective disorders, no. (%) | *1347 (0.6%)* | *1170 (0.6%)* | *177 (1.6%)* | *<0.001*** |
| Anxiety disorders, no. (%) | *3137 (1.5%)* | *2792 (1.4%)* | *345 (3.2%)* | *<0.001*** |
| Chronic obstructive pulmonary disease, no. (%) | *4421 (2.1%)* | *4048 (2.0%)* | *373 (3.5%)* | *<0.001*** |
| Year of surgery 2011-2014  (Compared to 2007-2010) , no. (%) | *122641 (58.0%)* | *116366 (57.9%)* | *6275 (58.4%)* | *0.36*** |
| **Type of surgery, no. (%)** |  |  |  |  |
| Non-elective surgery, no. (%) | *60629 (28.7%)* | *56902 (28.3%)* | *3727 (34.7%)* | *<0.001*** |
| Cancer surgery, no. (%) | *42994 (20.3%)* | *40335 (20.1%)* | *2659 (24.7%)* | *<0.001*** |
| Neuro, no. (%) | *14426 (6.8%)* | *12807 (6.4%)* | *1619 (15.1%)* | *<0.001*** |
| Endocrine, no. (%) | *6725 (3.2%)* | *6452 (3.2%)* | *273 (2.5%)* | *<0.001*** |
| Ophthalmic, no. (%) | *2927 (1.4%)* | *2843 (1.4%)* | *84 (0.8%)* | *<0.001*** |
| Ear Nose and Throat, no. (%) | *6232 (2.9%)* | *6056 (3.0%)* | *176 (1.6%)* | *<0.001*** |
| Oral and maxillofacial, no. (%) | *8537 (4.0%)* | *8198 (4.1%)* | *339 (3.2%)* | *<0.001*** |
| Thoracic (not including cardiac surgery) , no. (%) | *2160 (1.0%)* | *1992 (1.0%)* | *168 (1.6%)* | *<0.001*** |
| Breast, no. (%) | *10809 (5.1%)* | *10221 (5.1%)* | *588 (5.5%)* | *0.080*** |
| Abdominal, no. (%) | *41195 (19.5%)* | *39367 (19.6%)* | *1828 (17.0%)* | *<0.001*** |
| Urologic, no. (%) | *23198 (11.0%)* | *22132 (11.0%)* | *1066 (9.9%)* | *<0.001*** |
| Gynecologic, no. (%) | *16556 (7.8%)* | *15852 (7.9%)* | *704 (6.5%)* | *<0.001*** |
| Orthopedic, no. (%) | *64434 (30.4%)* | *61432 (30.6%)* | *3002 (27.9%)* | *<0.001*** |
| Vascular, no. (%) | *8693 (4.1%)* | *8089 (4.0%)* | *604 (5.6%)* | *<0.001*** |
| Dermatologic, no. (%) | *5722 (2.7%)* | *5424 (2.7%)* | *298 (2.8%)* | *0.65*** |
| Abbreviations: ASA = American Society of Anesthesiologists, SD = Standard Deviation. *ANOVA. ** Pearson’s Chi-squared test. | | | | |
