# Supplementary material for: Risk factors for new antidepressant use after surgery in Sweden: a nationwide, observational cohort study
Source: BJA Open. 2023 Jul 21;7:100218. doi: 10.1016/j.bjao.2023.100218 (PMC10457487; doi:10.1016/j.bjao.2023.100218)
Supplement: Multimedia component 2 [file mmc2.docx]

| **Supplemental Table 2** Patient and perioperative characteristics in relation to collection of antidepressant medication after major surgery | | | | | | | | | |
| --- | --- | --- | --- | --- | --- | --- | --- | --- | --- |
|  | ***Collected antidepressant prescription 0-365 days post-surgery*** | | | | | | | | |
|  | **Neurosurgery** | | | **Vascular surgery** | | | **Thoracic surgery (no heart surgery)** | | |
|  | ***No***  ***(n = 12807)*** | ***Yes***  ***(n = 1619)*** | ***P-value*** | ***No***  ***(n = 8089)*** | ***Yes***  ***(n = 604)*** | ***P-value*** | ***No***  ***(n = 1992)*** | ***Yes***  ***(n = 168)*** | ***P-value*** |
| **Background characteristics** |  |  |  |  |  |  |  |  |  |
| Age, years, mean ± SD | *57.5 (17.7)* | *57.6 (16.3)* | *0.91* | *66.4 (14.5)* | *67.5 (14.8)* | *0.064* | *54.1 (19.4)* | *59.4 (17.2)* | *<0.001** |
| Female sex, no. (%) | *5425 (42.4%)* | *758 (46.8%)* | *<0.001* | *3098 (38.3%)* | *279 (46.2%)* | *<0.001* | *760 (38.2%)* | *82 (48.8%)* | *0.007*** |
| ASA Classification, no. (%) |  |  | *<0.001* |  |  | *<0.001* |  |  | *<0.001*** |
| ASA1 | *2355 (18.4%)* | *165 (10.2%)* |  | *1228 (15.2%)* | *67 (11.1%)* |  | *406 (20.4%)* | *11 (6.5%)* |  |
| ASA2 | *4903 (38.3%)* | *462 (28.5%)* |  | *2979 (36.8%)* | *182 (30.1%)* |  | *740 (37.1%)* | *50 (29.8%)* |  |
| ASA3 | *5159 (40.3%)* | *814 (50.3%)* |  | *3588 (44.4%)* | *318 (52.6%)* |  | *766 (38.5%)* | *83 (49.4%)* |  |
| ASA4 | *390 (3.0%)* | *178 (11.0%)* |  | *294 (3.6%)* | *37 (6.1%)* |  | *80 (4.0%)* | *24 (14.3%)* |  |
| Heart disease, no. (%) | *3251 (25.4%)* | *437 (27.0%)* | *0.16* | *3839 (47.5%)* | *331 (54.8%)* | *<0.001* | *495 (24.8%)* | *46 (27.4%)* | *0.46*** |
| Chronic kidney disease, no. (%) | *110 (0.9%)* | *13 (0.8%)* | *1.00* | *766 (9.5%)* | *43 (7.1%)* | *0.059* | *25 (1.3%)* | *0 (0.0%)* | *0.25*** |
| Diabetes Mellitus, no. (%) | *715 (5.6%)* | *93 (5.7%)* | *0.77* | *948 (11.7%)* | *95 (15.7%)* | *0.005* | *106 (5.3%)* | *11 (6.5%)* | *0.48*** |
| Peripheral vascular disease, no. (%) | *327 (2.6%)* | *35 (2.2%)* | *0.40* | *2707 (33.5%)* | *215 (35.6%)* | *0.28* | *66 (3.3%)* | *5 (3.0%)* | *1.00*** |
| Cerebrovascular disease, no. (%) | *1023 (8.0%)* | *146 (9.0%)* | *0.16* | *688 (8.5%)* | *72 (11.9%)* | *0.006* | *41 (2.1%)* | *8 (4.8%)* | *0.051*** |
| Cognitive disease, no. (%) | *203 (1.6%)* | *32 (2.0%)* | *0.25* | *66 (0.8%)* | *12 (2.0%)* | *0.011* | *12 (0.6%)* | *2 (1.2%)* | *0.30*** |
| Substance abuse disorder, no. (%) | *168 (1.3%)* | *32 (2.0%)* | *0.041* | *138 (1.7%)* | *23 (3.8%)* | *<0.001* | *38 (1.9%)* | *6 (3.6%)* | *0.15*** |
| Miscellaneous psychiatric disorders, no. (%) | *266 (2.1%)* | *17 (1.1%)* | *0.004* | *65 (0.8%)* | *5 (0.8%)* | *0.82* | *50 (2.5%)* | *4 (2.4%)* | *1.00*** |
| Affective disorders, no. (%) | *85 (0.7%)* | *23 (1.4%)* | *0.002* | *47 (0.6%)* | *9 (1.5%)* | *0.014* | *19 (1.0%)* | *4 (2.4%)* | *0.098*** |
| Anxiety disorders, no. (%) | *207 (1.6%)* | *34 (2.1%)* | *0.15* | *87 (1.1%)* | *16 (2.6%)* | *0.002* | *34 (1.7%)* | *8 (4.8%)* | *0.014*** |
| Chronic obstructive pulmonary disease, no. (%) | *227 (1.8%)* | *36 (2.2%)* | *0.20* | *439 (5.4%)* | *39 (6.5%)* | *0.27* | *114 (5.7%)* | *15 (8.9%)* | *0.12*** |
| Non-elective surgery, no. (%) | *3465 (27.1%)* | *624 (38.5%)* | *<0.001* | *1337 (16.5%)* | *162 (26.8%)* | *<0.001* | *516 (25.9%)* | *71 (42.3%)* | *<0.001*** |
| Cancer surgery, no. (%) | *1162 (9.1%)* | *163 (10.1%)* | *0.20* | *2637 (32.6%)* | *149 (24.7%)* | *<0.001* | *638 (32.0%)* | *52 (31.0%)* | *0.80*** |
| Abbreviations: ASA = American Society of Anesthesiologists, SD = Standard Deviation. *ANOVA. ** Fisher's exact test. | | | | | | | | | |
