# Supplementary material for: Risk factors for new antidepressant use after surgery in Sweden: a nationwide, observational cohort study
Source: BJA Open. 2023 Jul 21;7:100218. doi: 10.1016/j.bjao.2023.100218 (PMC10457487; doi:10.1016/j.bjao.2023.100218)
Supplement: Multimedia component 3 [file mmc3.docx]

| **Supplemental Table 3.** Variables independently associated with antidepressant use 0-365d after major surgery. Multivariable model 1: All characteristics variables in table 1 eligible for inclusion except ASA classification. | | |
| --- | --- | --- |
|  | **Crude odds ratio**  **(95% CI)** | **Multivariable adjusted odds ratio**  **(95% CI)** |
| **Neurosurgery** |  |  |
| Age (years)* | p <0.0001 | p <0.0001 |
| 18 | 1.0 (Reference ) | 1.0 (Reference) |
| 30 | 1.22 (1.12 - 1.33) | 1.25 (1.13 - 1.34) |
| 40 | 1.43 (1.23 - 1.67) | 1.45 (1.24 - 1.70) |
| 50 | 1.61 (1.31 - 1.98) | 1.64 (1.33 - 2.02) |
| 60 | 1.64 (1.30 - 2.06) | 1.65 (1.31 - 2.08) |
| 70 | 1.46 (1.17 - 1.81) | 1.45 (1.16 - 1.80) |
| 80 | 1.21 (0.98 - 1.48) | 1.17 (0.95 - 1.44) |
| 90 | 0.99 (0.79 - 1.24) | 0.93 (0.74 - 1.17) |
| Female sex | 1.20 (1.08 - 1.33) | 1.24 (1.12 - 1.38) |
| Miscellaneous psychiatric disorders | 0.50 (0.31 - 0.82) | 0.50 (0.30 - 0.83) |
| Affective disorders | 2.16 (1.36 - 3.43) | 2.23 (1.39 - 3.59) |
| Non-elective surgery | 1.69 (1.52 - 1.88) | 1.77 (1.59 - 1.97) |
| **Vascular surgery** |  |  |
| Female sex | 1.38 (1.17 - 1.63) | 1.49 (1.26 - 1.77) |
| Diabetes mellitus | 1.41 (1.12 - 1.77) | 1.34 (1.06 - 1.70) |
| Cognitive disease | 2.46 (1.32 - 4.58) | 1.99 (1.05 - 3.75) |
| Affective disorders | 2.59 (1.26 - 5.31) | 2.22 (1.06 - 4.63) |
| Anxiety disorders | 2.50 (1.46 - 4.29) | 2.14 (1.23 - 3.73) |
| Substance abuse disorder | 2.28 (1.46 - 3.57) | 2.09 (1.32 - 3.30) |
| Non-elective surgery | 1.85 (1.53 - 2.24) | 1.73 (1.41 - 2.11) |
| Cancer surgery | 0.68 (0.56 - 0.82) | 0.76 (0.62 - 0.94) |
| **Thoracic surgery (not including cardiac surgery)** |  |  |
| Age (years)* |  | p <0.01 |
| 18 | 1.0 (Reference ) | 1.0 (Reference) |
| 30 | 1.30 (0.99 - 1.71) | 1.31 (0.99 - 1.73) |
| 40 | 1.60 (1.00 - 2.57) | 1.61 (0.99 - 2.60) |
| 50 | 1.93 (1.06 - 3.51) | 1.92 (1.04 - 3.55) |
| 60 | 2.23 (1.19 - 4.19) | 2.19 (1.15 - 4.20) |
| 70 | 2.47 (1.38 - 4.43) | 2.37 (1.29 - 4.36) |
| 80 | 2.68 (1.50 - 4.77) | 2.51 (1.38 - 4.57) |
| 90 | 2.90 (1.49 - 5.65) | 2.64 (1.34 - 5.23) |
| Female sex | 1.55 (1.13 - 2.12) | 1.67 (1.20 - 2.32) |
| Cerebrovascular disease | 2.38 (1.10 - 5.16) | 2.30 (1.03 - 5.13) |
| Anxiety disorders | 2.88 (1.31 - 6.32) | 2.87 (1.26 - 6.51) |
| Non-elective surgery | 2.09 (1.52 - 2.89) | 2.32 (1.66 - 3.23) |
| *The nonlinear association between age and the outcome was modeled using restricted cubic splines. The odds ratios and confidence intervals according to the age categories were calculated based on the spline coefficients. The p value was calculated using Wald’s test. Abbreviations: ASA = American Society of Anesthesiologists, CI = Confidence interval. | | |
