# Supplementary material for: Risk factors for new antidepressant use after surgery in Sweden: a nationwide, observational cohort study
Source: BJA Open. 2023 Jul 21;7:100218. doi: 10.1016/j.bjao.2023.100218 (PMC10457487; doi:10.1016/j.bjao.2023.100218)
Supplement: Multimedia component 4 [file mmc4.docx]

| **Supplemental Table 4.** Variables independently associated with antidepressant use 0-365d after major surgery. Multivariable model 2: Variables age, sex, ASA classification, non-elective surgery, and cancer surgery eligible for inclusion. | | |
| --- | --- | --- |
|  | **Crude odds ratio**  **(95% CI)** | **Multivariable adjusted odds ratio**  **(95% CI)** |
| **Neurosurgery** |  |  |
| ASA classification | p <0.0001 | p < 0.0001 |
| ASA1 | 1.0 (Reference) | 1.0 (Reference) |
| ASA2 | 1.34 (1.12 - 1.62) | 1.46 (1.21 - 1.77) |
| ASA3 | 2.25 (1.89 - 2.68) | 2.34 (1.95 - 2.80) |
| ASA4 | 6.51 (5.14 - 8.26) | 6.05 (4.73 - 7.74) |
| Age (years)* | p <0.0001 | p <0.0001 |
| 18 | 1.0 (Reference ) | 1.0 (Reference) |
| 30 | 1.22 (1.12 - 1.33) | 1.15 (1.06 - 1.25) |
| 40 | 1.43 (1.23 - 1.67) | 1.29 (1.10 - 1.50) |
| 50 | 1.61 (1.31 - 1.98) | 1.37 (1.11 - 1.69) |
| 60 | 1.64 (1.30 - 2.06) | 1.32 (1.05 - 1.67) |
| 70 | 1.46 (1.17 - 1.81) | 1.11 (0.89 - 1.39) |
| 80 | 1.21 (0.98 - 1.48) | 0.87 (0.70 - 1.08) |
| 90 | 0.99 (0.79 - 1.24) | 0.67 (0.53 - 0.85) |
| Female sex | 1.20 (1.08 - 1.33) | 1.23 (1.11 - 1.37) |
| Non-elective surgery | 1.69 (1.52 - 1.88) | 1.43 (1.28 - 1.60) |
| **Vascular surgery** |  |  |
| ASA classification | p < 0.0001 | p < 0.0001 |
| ASA1 | 1.0 (Reference) | 1.0 (Reference) |
| ASA2 | 1.12 (0.84 - 1.49) | 1.16 (0.87 - 1.55 |
| ASA3 | 1.62 (1.24 - 2.13) | 1.66 (1.26 - 2.19) |
| ASA4 | 2.31 (1.51 - 3.51) | 2.17 (1.41 - 3.35) |
| Female sex | 1.38 (1.17 - 1.63) | 1.51 (1.28 - 1.79) |
| Non-elective surgery | 1.85 (1.53 - 2.24) | 1.69 (1.39 - 2.05) |
| **Thoracic surgery (not including cardiac surgery)** |  |  |
| ASA classification | p < 0.0001 | p < 0.0001 |
| ASA1 | 1.0 (Reference) | 1.0 (Reference) |
| ASA2 | 2.49 (1.28 - 4.84) | 2.87 (1.46 - 5.63) |
| ASA3 | 4.00 (2.11 - 7.59) | 4.41 (2.31 - 8.43) |
| ASA4 | 11.07 (5.22 - 23.51) | 9.24 (4.32 - 19.76) |
| Female sex | 1.55 (1.13 - 2.12) | 1.63 (1.17 - 2.26) |
| Non-elective surgery | 2.09 (1.52 - 2.89) | 2.25 (1.59 - 3.17) |
| *The nonlinear association between age and the outcome was modeled using restricted cubic splines. The odds ratios and confidence intervals according to the age categories were calculated based on the spline coefficients. The p value was calculated using Wald’s test. Abbreviations: ASA = American Society of Anesthesiologists, CI = Confidence interval. | | |
