# Supplementary material for: Risk factors for new antidepressant use after surgery in Sweden: a nationwide, observational cohort study
Source: BJA Open. 2023 Jul 21;7:100218. doi: 10.1016/j.bjao.2023.100218 (PMC10457487; doi:10.1016/j.bjao.2023.100218)
Supplement: Multimedia component 5 [file mmc5.docx]

| **Supplemental table 5.** Definition of variables. | |
| --- | --- |
| **Background characteristic** | **International classification of diseases revision 10** |
| Heart disease | I05 to I08, I10 to I15, I20 to I22, I24 to I28, I34 to I37, I42, I44 to I50. (Diagnosis within 5 years) |
| Chronic kidney disease | N18 (Diagnosis within 5 years) |
| Diabetes Mellitus | E10, E12 (Diagnosis within 5 years)  E11 (Diagnosis within 1 year) |
| Peripheral vascular disease | I70-I79 (Diagnosis within 5 years) |
| Cerebrovascular disease | I60 to I63 (Diagnosis within 5 years)  I64 to I 66 (Diagnosis within 1 year)  I67 to I69 (Diagnosis within 5 years) |
| Cognitive disease | F00 to F08 (Diagnosis within 5 years) |
| Substance abuse disorder | F10 to F19 (Diagnosis within 1 year) |
| Miscellaneous psychiatric disorders | F20 to F29, F50 to F99 (Diagnosis within 5 years) |
| Affective disorders | F30 to F39 (Diagnosis within 5 years) |
| Anxiety disorders | F40 to F48 (Diagnosis within 5 years) |
| Chronic obstructive pulmonary disease | J44 (Diagnosis within 5 years) |
